# Machine learning approaches for the identification of real-world predictors of mepolizumab response

**DOI:** 10.1371/journal.pone.0362402

**Published:** 2026-11-13

**Authors:** Koyo Usuba, Lingjiao Zhang, Xinyang Liu, Tim Tian Yu Han, Natalie Nightingale, Ali Tehrani, Peter Howarth, Rafael Alfonso-Cristancho

**Affiliations:** 1 GSK, Mississauga, Ontario, Canada; 2 GSK, Collegeville, Pennsylvania, United States of America; 3 IQVIA Solutions Canada Inc., Kirkland, Quebec, Canada; 4 GSK, Brentford, United Kingdom; University of Connecticut Health Center, UNITED STATES OF AMERICA

## Abstract

**Background:**

Mepolizumab improves control and reduces clinically significant exacerbations in severe asthma patients. Further research is needed to understand factors influencing patient response.

**Objective:**

Identify predictors of response to mepolizumab using machine learning techniques.

**Methods:**

Thirty clinically relevant features from the REALITI-A dataset were analyzed. Response to mepolizumab was defined as change in clinically significant exacerbations at Year 1 post-exposure, being free from clinically significant exacerbations at Year 1, and being free from clinically significant exacerbations at both Year 1 and Year 2 post-exposure for primary, secondary, and exploratory analyses, respectively. Linear/logistic regression, random forest, and XGBoost models were tested. The best-performing model based on R^2^ or classification accuracy was used for feature selection. A SHapley Additive exPlanations post-hoc analysis was conducted.

**Results:**

The primary and secondary analyses included 685 patients; the exploratory analysis included 529 patients. Using XGBoost for the primary analysis, higher pre-exposure clinically significant exacerbations rate, lower body mass index, total immunoglobulin E, lower baseline activity impairment, and lower baseline Asthma Control Questionnaire-5 score were top predictors. For the secondary analysis, the random forest model identified lower pre-exposure clinically significant exacerbations rate, residency outside the United Kingdom, and lower baseline Asthma Control Questionnaire-5 score, body mass index, and baseline activity impairment as top predictors. For the exploratory analysis, XGBoost identified residency outside the United Kingdom, and lower pre-exposure clinically significant exacerbations rate, baseline Asthma Control Questionnaire-5 score, eosinophil levels, and body mass index as top predictors.

**Conclusion:**

Machine learning models identified key predictors of response to mepolizumab, which could guide clinical decision-making and improve patient outcomes in severe asthma patients.

## Introduction

Asthma is a widely prevalent chronic respiratory condition affecting individuals of all ages, with an estimated 262 million cases worldwide in 2019 [1,2]. While most asthma cases are mild, about 5%–10% of patients are affected by severe asthma [3,4]. The 2024 Global Initiative for Asthma (GINA) defines severe asthma as asthma that remains uncontrolled despite high-intensity treatment, such as high-dose inhaled corticosteroids (ICS) combined with additional controller medications [5]. Many patients also require maintenance or episodic oral corticosteroids (OCS) following exacerbations [5]. However, prolonged OCS use is associated with substantial morbidity, including osteoporosis, hypertension, diabetes, and an increased risk of infection, which can significantly impair patients’ quality of life (QoL) [6–9]. Therefore, alternative effective treatment options with an improved side effect profile are necessary for the management of severe asthma.

Mepolizumab, an anti-IL-5 monoclonal antibody, has emerged as a promising therapeutic option for severe asthma [7,10]. Previous clinical studies have demonstrated that mepolizumab effectively reduces clinically significant exacerbations (CSE), decreases the maintenance OCS dosage, and improves lung function and QoL [11–14]. Moreover, a more recent multi-country, prospective, observational study, REALITI-A, demonstrated the real-world effectiveness and safety of mepolizumab across seven countries [15]. Analysis performed with data collected from the REALITI-A study demonstrated that mepolizumab can reduce the rate of CSE in patients with severe asthma, and reduce the need for maintenance OCS and episodic systemic corticosteroids (SCS) [16]. However, response to mepolizumab can vary across patients, with the factors predicting optimal response to mepolizumab not well understood. A better understanding of predictors of mepolizumab response in patients with severe asthma would be invaluable in supporting physician decision-making and improving treatment selection and management of patients with severe asthma in the real-world. While subgroup analysis can identify key factors associated with improved clinical outcomes, these approaches are not sophisticated enough to model an extensive list of candidate factors, nor their potentially complex interactions, and thus are not able to provide robust and precise results on predictors of response.

The application of machine learning (ML) methodologies to large healthcare datasets to improve clinical decision-making and disease management is on the rise. Specifically, ML approaches have also been utilized to predict patient response to treatments based on data collected from complex datasets. One such study used data from randomized clinical trials involving patients with rheumatoid arthritis and leveraged ML methods such as the least absolute shrinkage and selection operator (LASSO) and random forest methods to identify predictors of response to methotrexate [17]. Applied tree-based ML methodologies have also been used to predict response to ICS in patients with asthma using data from a European Ancestry database [18]. While these studies demonstrate the feasibility of using ML methodologies to identify predictors of treatment response, there have not yet been any studies using ML to identify predictors of response to mepolizumab in patients with severe asthma. As such, the aim of this study was to identify predictors of response to mepolizumab using ML methodologies on a healthcare dataset. Given the expectation that demographics, past treatments, and clinical factors related to the patient’s medical history and asthma severity would be important candidate predictors to consider, we sought to use a dataset that captured such information in a meaningful number of patients with severe asthma. With these considerations, the REALITI-A study was identified as an appropriate dataset for this analysis given its broad capture of clinically relevant information, and adequate sample size. Although other datasets may have captured a larger sample size, the capture of clinically relevant candidate predictors would have been limited.

## Methodology

### Data source

Data from participants of the REALITI-A study who provided consent were included. The REALITI-A study [15,19] captured information from patient medical records and electronic case report forms (electronic health records and administrative claims data) between December 2016 and October 2019. This prospective observational cohort study enrolled 822 patients from across seven countries, who were diagnosed with asthma and newly prescribed mepolizumab by their treating physician (Fig 1). Data were accessed for the current analysis from November 2023 to March 2024. All data were deidentified; authors did not have access to any participant-identifying information during the analysis.

**Fig 1:**
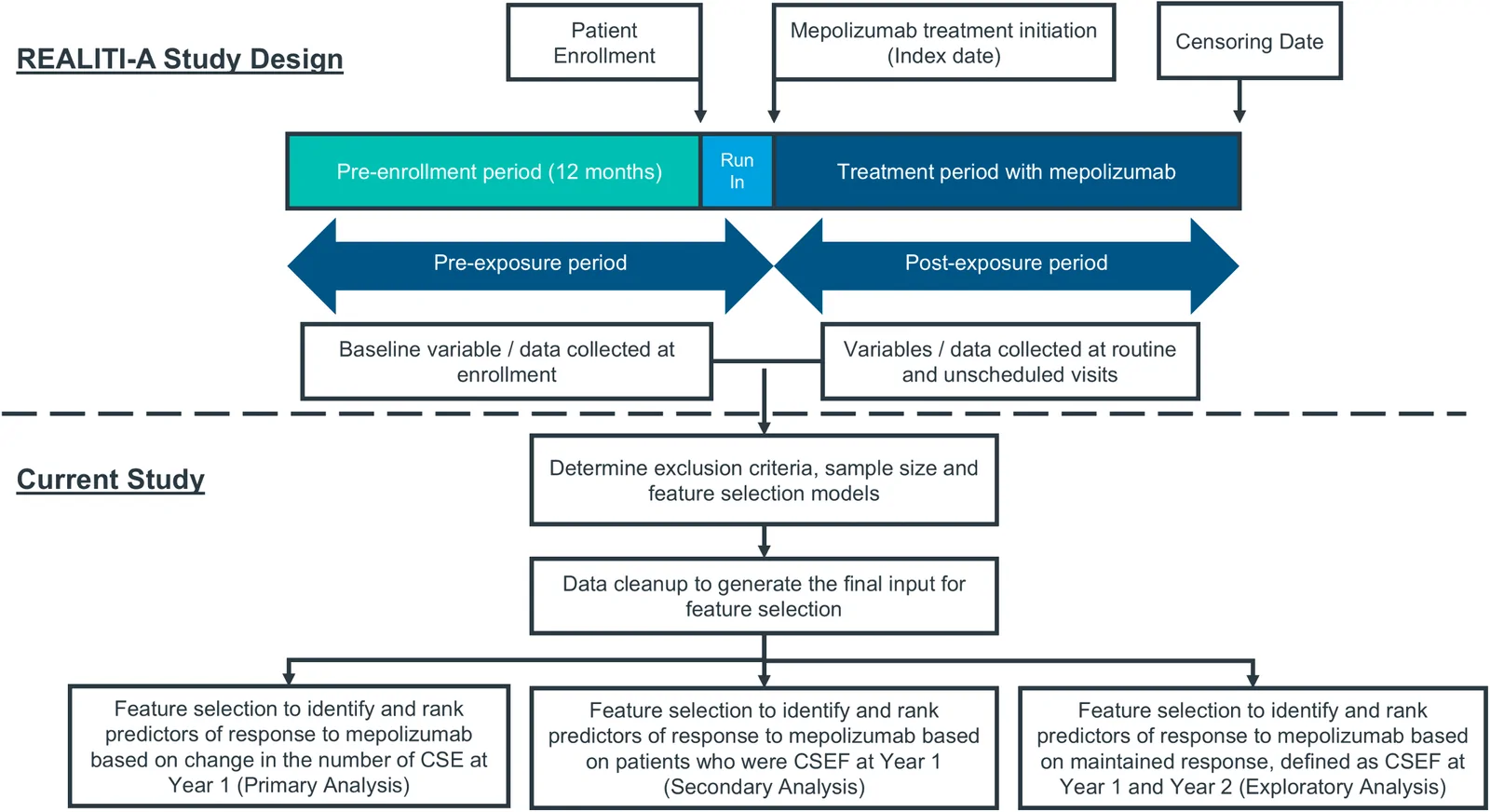
Study design and key time periods.

Patients were excluded from Year 1 analysis if they met any of the following criteria: discontinued from the REALITI-A study (e.g., due to death or withdrawal of consent) before Year 1 post-exposure to mepolizumab; enrolled in Spain or Germany and withdrew consent in Year 1 or Year 2 post-exposure to mepolizumab; or had missing values for the number of exacerbations (EXBNUM) variable at Year 1 post-exposure to mepolizumab. Additionally, participants who discontinued from the REALITI-A study before Year 2 post-exposure to mepolizumab and those with missing values for EXBNUM variable at Year 2 post-exposure were excluded from all analyses requiring Year 2 post-exposure data.

### Variables

A total of 30 clinically relevant features were collected from REALITI-A study for feature selection. These features were encoded into 122 variables based on clinical relevance, including the top 29 medications and top 23 comorbidities among eligible patients. A complete list of variables included for feature selection is in S1-S3 Tables.

### Measurement of outcomes

The primary endpoint was change in CSE, defined as the difference in the number of CSE events during the one-year immediately following mepolizumab initiation (i.e., Year 1 post-exposure period) versus the pre-exposure period. As described in previous publications on REALITI-A [19], CSE events were defined as a deterioration in asthma symptoms requiring SCS, and/or deterioration requiring hospitalization or an emergency department visit. The secondary endpoint, free from CSE (CSEF) at Year 1, was defined as the absence of any CSE events from the index date to the end of Year 1 post-exposure. The exploratory endpoint, maintained response, was defined as being CSEF at both Year 1 and Year 2 post-exposure to mepolizumab (Fig 1).

### Statistical analysis

Patient demographics and clinical characteristics were reported for the pre-exposure period. Descriptive statistics were presented as mean, standard deviation (SD), median, interquartile range (IQR), minimum, and maximum for continuous variables, and frequency and proportion for categorical variables.

### Machine learning techniques

For each study endpoint, prediction algorithms were built and compared using the following steps: data preparation, training and evaluation of the model, and final interpretation of feature rankings. An initial data pre-processing step was performed to make the features suitable for ML models. Continuous features were scaled using Z-score normalization. Nominal data were transformed using one-hot encoding, while ordinal data were processed using label encoding and subsequently normalized as continuous features. Missing values were imputed using the k-Nearest Neighbors (kNN) method.

A total of three models, logistic/linear regression, random forest, and extreme gradient boosting (XGBoost) [20], were tested for each outcome analysis. More specifically, linear regression was used for change in CSE, and logistic regression was used for CSEF at Year 1 post-exposure and CSEF at both Year 1 and Year 2 post-exposure. Five-fold cross-validation was performed during the feature selection process. In brief, the cross-validation algorithm generated new models by reshuffling the training and testing datasets until all data were used as part of the test dataset. Each model test iteration produced a specific performance metric, and the final classification performance of the model was the average performance obtained. This was based on R^2^ for change in CSE and classification accuracy for CSEF at Year 1 post-exposure and CSEF at both Year 1 and Year 2 post-exposure. The best-performing model for each analysis was used for feature selection and importance measures.

Following model training, SHapley Additive exPlanations (SHAP) [21] post-hoc analyses were used to interpret the model outputs. Shapley values were calculated for each feature and approximated the individual contribution of each feature by estimating the model output in the absence of the specific feature and comparing it to all the models with the features included. The mean absolute SHAP value for each feature across the entire dataset was calculated to quantify the contribution magnitude of each feature to the predicted outcome. Features with higher mean absolute SHAP values were more influential. A waterfall plot showing the directionality of the top 10 features, as well as the aggregate SHAP value of the remaining features for one randomly selected patient, was generated for each analysis. The SHAP values were normalized in order to be graphed on the same axis. The f(x) values shown in waterfall plots represent the predicted value of the outcome for the randomly selected patient while the E[f(X)] value is the average value of the outcome for all patients in the training data.

## Results

### Patient disposition

A total of 685 participants from REALITI-A met the eligibility criteria for this study and were included in the analyses of the primary and secondary objectives. For the exploratory analysis, 156 participants were excluded due to discontinuation prior to Year 2, resulting in a final cohort of 529 participants for the exploratory objective (Table 1).

**Table 1:** Demographic characteristics of patients for each primary, secondary and exploratory analysis.

Characteristics	Primary and secondary analyses (N = 685)	Exploratory analysis (N = 529)
**Sex, n (%)**		
n	685	529
Female	436 (63.65)	336 (63.52)
Male	249 (36.35)	193 (36.48)
**Age (years)** ^**a**^		
n	685	529
mean (SD)	54.78 (13.25)	55.19 (13.19)
**Age group (years), n (%)**		
		
18-64	519 (75.77)	393 (74.29)
65-84	165 (24.09)	135 (25.52)
≥85	1 (0.15)	1 (0.19)
**Ethnicity, n (%)**		
n	685	529
Hispanic or Latino	98 (14.31)	81 (15.31)
Not Hispanic or Latino	587 (85.69)	448 (84.69)
**Race, n (%)**		
n	685	529
Asian, Native Hawaiian or Other Pacific Islander	24 (3.50)	15 (2.84)
Black or African American	19 (2.77)	12 (2.27)
White or Caucasian	635 (92.70)	497 (93.95)
Other	4 (0.58)	3 (0.57)
Multiple	2 (0.29)	1 (0.19)
Missing	1 (0.15)	1 (0.19)
**BMI (kg/m**^**2**^)		
n	684	528
Mean (SD)	28.92 (7.31)	28.57 (7.20)
**Geographic location, n (%)**		
n	685	529
Belgium	34 (4.96)	29 (5.48)
Germany	66 (9.64)	48 (9.07)
Italy	218 (31.82)	189 (35.73)
Spain	81 (11.82)	67 (12.67)
United Kingdom	162 (23.65)	120 (22.68)
Canada	45 (6.57)	25 (4.73)
United States	79 (11.53)	51 (9.64)
**Smoking status, n (%)**		
n	685	529
Never smoked	422 (61.61)	333 (62.95)
Current smoker	17 (2.48)	10 (1.89)
Former smoker	241 (35.18)	182 (34.40)
Missing	5 (0.73)	4 (0.76)
**Asthma duration (years)**		
n	673	518
Mean (SD)	19.49 (15.55)	19.06 (15.48)
**Primary reason for treating with mepolizumab, n (%)**		
n	685	529
Reduce exacerbations	271 (39.56)	208 (39.32)
Reduce burden of oral corticosteroids	154 (22.48)	130 (24.57)
Improve asthma symptoms	149 (21.75)	110 (20.79)
Improve quality of life	50 (7.30)	41 (7.75)
Improve lung function	8 (1.17)	7 (1.32)
Missing	53 (7.74)	33 (6.24)
**Total CSE**		
n	685	529
Mean (SD)	4.36 (4.00)	4.31 (3.81)
Median (min, max)	3 (0, 34)	3 (0, 34)
Q1, Q3	2, 5	2, 5
0, n (%)	44 (6.42)	30 (5.67)
1, n (%)	86 (12.55)	65 (12.29)
2, n (%)	133 (19.42)	108 (20.42)
3, n (%)	117 (17.08)	92 (17.39)
4, n (%)	71 (10.36)	56 (10.59)
5, n (%)	64 (9.34)	47 (8.88)
>5, n (%)	170 (24.82)	131 (24.76)
**Blood eosinophils (GI/L)**		
n	509	391
Geometric mean (SD log)	0.35 (1.27)	0.38 (1.21)
Median (min, max)	0.44 (0, 7.4)	0.48 (0, 7.4)
Q1, Q3	0.25, 0.8	0.28, 0.82
<0.15 GI/L, n (%)	79 (15.52)	55 (14.07)
0.15 – < 0.3 GI/L, n (%)	66 (12.97)	47 (12.02)
0.3 – < 0.5 GI/L, n (%)	127 (24.95)	98 (25.06)
≥0.5 GI/L, n (%)	237 (46.56)	191 (48.85)
**ACQ-5 score** ^**+**^		
n	651	504
Mean (SD)	2.8 (1.3)	2.8 (1.3)
Median (min, max)	3 (0, 6)	2.8 (0, 6)
Q1, Q3	2, 3.7	1.95, 3.6
<0.75, n (%)	53 (8.14)	42 (8.33)
0.75 – < 1.50, n (%)	62 (9.52)	56 (11.11)
≥1.50, n (%)	536 (82.33)	406 (80.56)
Medical conditions*		
Class: Any, n (%)	647 (94.45)	502 (94.90)
**Class: Asthma disorders, n (%)**		
Any	564 (82.34)	438 (82.80)
Hay fever	347 (50.66)	273 (51.61)
Chronic sinusitis	289 (42.19)	220 (41.59)
Nasal polyp	278 (40.58)	219 (41.40)
Drug hypersensitivity	223 (32.55)	167 (31.57)
Nasal polypectomy	204 (29.78)	161 (30.43)
Atopic dermatitis	72 (10.51)	49 (9.26)
Anaphylaxis	52 (7.59)	38 (7.18)
**Class: Other comorbidities, n (%)**		
Gastroesophageal reflux disease	264 (38.54)	205 (38.75)
Diabetes	82 (11.97)	55 (10.40)
Food allergy	80 (11.68)	54 (10.21)
Cataract	65 (9.49)	53 (10.02)
Chronic obstructive pulmonary disease	60 (8.76)	40 (7.56)
Pneumonia	58 (8.47)	44 (8.32)
Oropharyngeal candidiasis	48 (7.01)	34 (6.43)
Churg Strauss syndrome	35 (5.11)	30 (5.67)
Glaucoma	25 (3.65)	19 (3.59)
Neuropathy	24 (3.50)	14 (2.65)
Hypereosinophilic syndrome	14 (2.04)	10 (1.89)
Peptic ulcer	14 (2.04)	9 (1.70)
Vasculitis	13 (1.90)	10 (1.89)
Angina pectoris	10 (1.46)	7 (1.32)
Myocardial infarction	10 (1.46)	9 (1.70)
Eosinophilic esophagitis	5 (0.73)	4 (0.76)

ACQ-5, Asthma Control Questionnaire-5; BMI, body mass index; CSE, clinically significant exacerbations; GI/L, 10^9^ cells per liter; Q1, first quartile; Q3, third quartile; SD, standard deviation

a Age was imputed when full date of birth was not provided.

+The ACQ-5 is scored on a 7-point scale from 0 = no impairment to 6 = maximum impairment. If a participant did not complete two or more of the five questions at a visit, then their ACQ-5 score was set to missing at that visit

*In the ‘Any’ categories, participants were counted only once if they had any medical condition. The other categories are not mutually exclusive, wherein patients may be captured under multiple categories if they had multiple medical conditions of interest.

### Baseline demographic and clinical characteristics

Of the 685 patients included for the primary and secondary analyses, 63.65% were female, and 92.70% identified as White or Caucasian. The mean (SD) age of the cohort was 54.78 (13.25) years, with a mean (SD) body mass index (BMI) of 28.92 (7.31) kg/m^2^, and an average asthma duration of 19.49 (15.55) years. Most patients (61.61%) had never smoked, and the primary reason for initiating mepolizumab was to reduce exacerbations (39.56%). The study population was predominantly from Italy (31.82%) and the United Kingdom (UK) (23.65%), with patients from Germany, Spain, the United States, Belgium, and Canada accounting for the remaining 44.5% of patients, reflecting a broad multinational scope (Table 1). Patients in this group had a mean CSE frequency of 4.36 in the pre-exposure period, with 24.82% of patients reported having more than 5 CSEs in the pre-exposure period. The demographic and clinical characteristics of the 529 patients included in the exploratory analyses were similar to those of the overall cohort (Table 1). Furthermore, blood eosinophil levels were similar between groups, with a geometric mean of 0.35 GI/L (SD 1.27) and 0.38 GI/L (SD 1.21), and median levels of 0.44 and 0.48 GI/L, respectively. Additionally, 46.56% and 48.85% of participants exhibited eosinophil levels ≥0.5 GI/L. Asthma Control Questionnaire-5 (ACQ-5) scores demonstrated comparable asthma control among groups, with mean scores of 2.8 (SD 1.3), and a majority (≥1.50 ACQ-5) comprising 82.33% and 80.56% of each cohort (Table 1).

Additional clinical characteristics of patients included in the primary and secondary analyses and those in the exploratory analysis were also similar (S4 Table). For both groups, hay fever was the most common asthma comorbidity reported by 50.66% and 51.61% of patients in each primary, secondary analysis and exploratory analysis, respectively. Roughly 38% of patients in both groups reported gastroesophageal reflux disease.

### Primary analysis: Change in the frequency of CSE at Year 1 post-exposure

Of the three models evaluated for change in frequency of CSE at Year 1 post-exposure, the best-performing model was XGBoost, with an R^2^ of 0.83, followed by Linear Regression and Random Forest, both with an R^2^ of 0.81. XGBoost was therefore selected for feature selection for this analysis. Following cross-validation, the model returned an R^2^ value of 0.77, with an adjusted R^2^ of 0.71 (Table 2).

**Table 2:** Performance metrics of ML models used for each primary, secondary and exploratory analysis.

Primary Analysis	
Number of observations, n	685
Number of features, n	122
MAE, mean (SD)	1.14 (0.22)
MSE, mean (SD)	3.08 (1.42)
RMSE, mean (SD)	1.72 (0.36)
R^2^, mean (SD)	0.77 (0.05)
Adjusted R^2^, mean (SD)	0.71 (0.06)
**Secondary Analysis**	
Number of observations, n	685
Number of features, n	122
Accuracy, mean (SD)	0.69 (0.02)
Precision, mean (SD)	0.67 (0.02)
Recall, mean (SD)	0.80 (0.04)
f1, mean (SD)	0.73 (0.02)
AUC, mean (SD)	0.73 (0.03)
**Exploratory Analysis**	
Number of observations, n	529
Number of features, n	122
Accuracy, mean (SD)	0.68 (0.04)
Precision, mean (SD)	0.64 (0.04)
Recall, mean (SD)	0.64 (0.09)
f1, mean (SD)	0.64 (0.06)
AUC, mean (SD)	0.73 (0.04)
Log loss, mean (SD)	0.60 (0.03)

AUC, area under the curve; MAE, mean absolute error; MSE, mean squared error; RMSE, root mean squared error. Note: In python, the scoring objects all follow the convention that higher return values are better than lower return values, so metrics such as MAE, MSE, and RMSE all return the negated version of the metric.

XGBoost identified higher pre-exposure exacerbation rates of CSE, lower BMI, total serum immunoglobulin E (IgE) levels, lower baseline activity impairment (collected from Work Productivity and Activity Impairment Questionnaire), and lower baseline ACQ-5 score as the top five predictors of the change in the frequency of CSE. Several variables associated with concomitant medications, medication history and asthma duration were also identified as top predictors (Fig 2).

**Fig 2:**
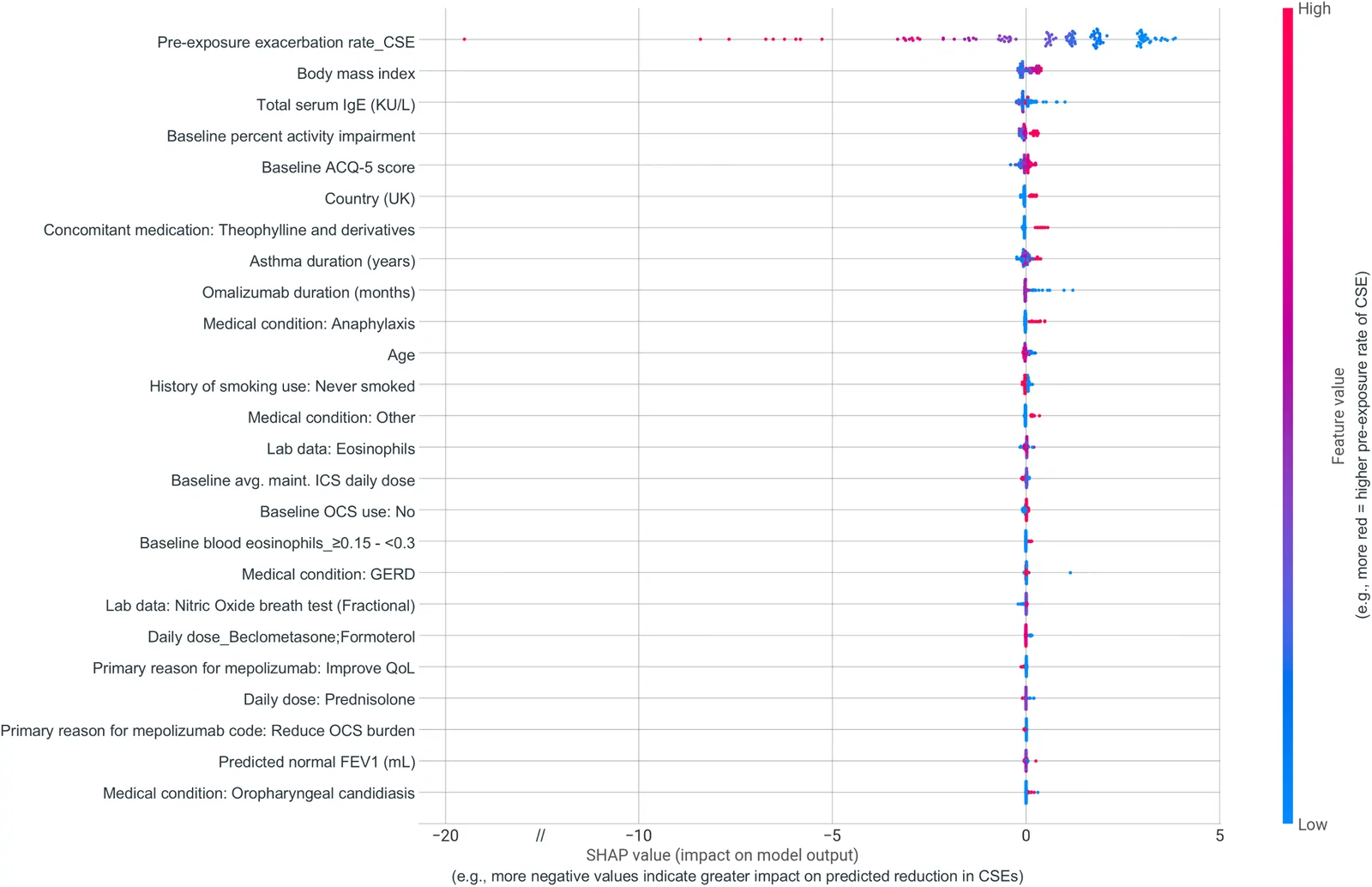
SHAP summary plot for the primary analysis. ACQ-5, Asthma Control Questionnaire-5; BMI, body mass index; CSE, clinically significant exacerbation; FEV1, forced expiratory volume in 1 second; GERD, gastroesophageal reflux disease; ICS, inhaled corticosteroids; IgE, immunoglobulin E; OCS, oral corticosteroids; QoL, quality of life; SHAP, SHapley Additive exPlanations; UK, United Kingdom.

A SHAP analysis was performed to delineate the directionality of the relationship between predictors and the outcome. The results suggest that higher pre-exposure CSE rates, lower BMI, lower baseline ACQ-5 score, and lower baseline activity impairment are associated with greater reductions in the frequency of CSE. While a top predictor, the directionality of total IgE as a predictor was inconclusive (Fig 2). A waterfall plot of one randomly selected patient is shown in Fig 3. Consistent with the overall SHAP results, the waterfall plot suggests that the model predicts that the pre-exposure rate of CSE would result in a reduction in the frequency of CSE (Fig 3).

**Fig 3:**
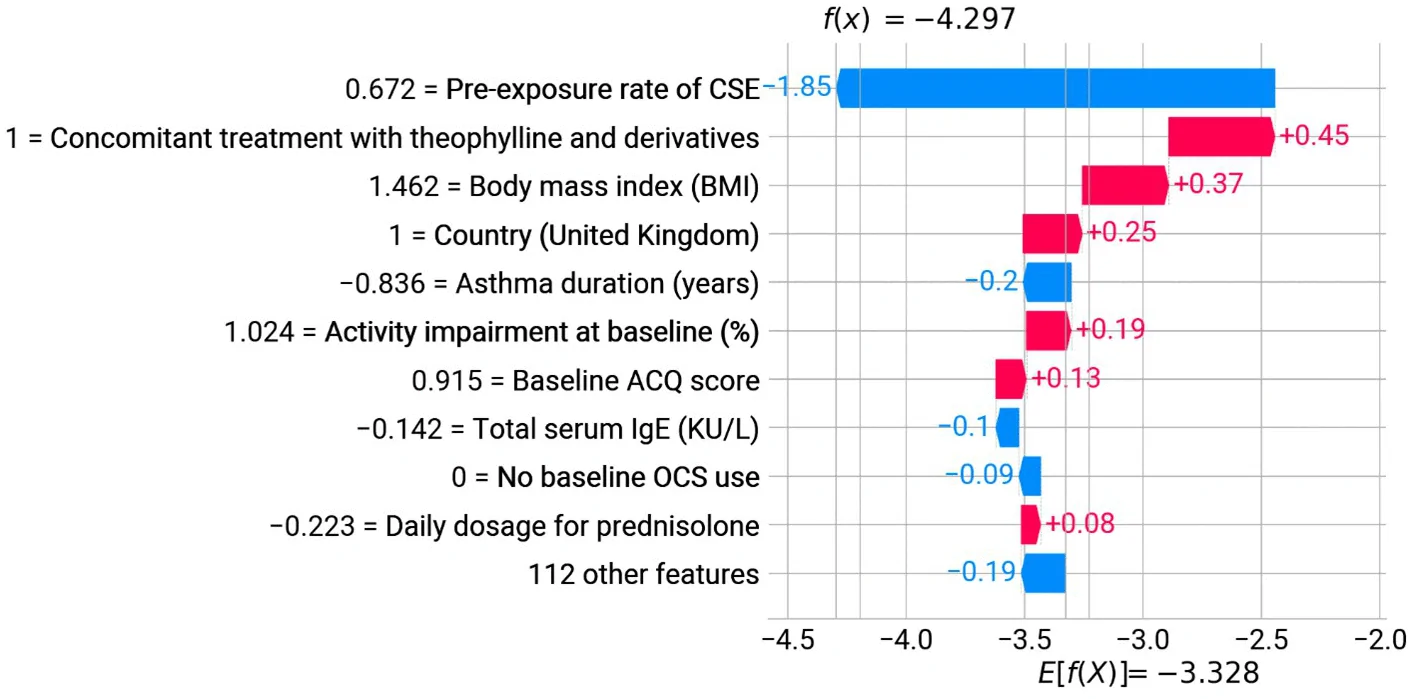
SHAP waterfall plot showing the output of the SHAP analysis for one randomly selected patient included in the primary analysis. SHAP values are indicated in the bar graphs. The values to the left of the feature names are normalized values of the feature for this randomly selected patient. ACQ-5, Asthma Control Questionnaire-5; BMI, body mass index; CSE, clinically significant exacerbation; IgE, immunoglobulin E; OCS, oral corticosteroids.

### Secondary analysis: CSEF at Year 1 post-exposure

To explore predictors of being CSEF at Year 1 post-exposure, Logistic Regression, Random Forest, and XGBoost were compared. The Logistic Regression model had an accuracy of 62.8%; both Random Forest and XGBoost had an accuracy of 69.3%. Even though both tree-based methodologies had similar accuracy, Random Forest had a more balanced accuracy. As such, Random Forest was used for feature selection. Following cross-validation, the model still retained an accuracy of 69% with an area under the curve (AUC) of 0.73, which is indicative of model stability (Table 2).

Feature selection with Random Forest and SHAP post-hoc analysis suggests that lower pre-exposure rate of CSE was a top predictor of being CSEF at Year 1, followed by residence outside of the UK, lower baseline ACQ-5 score, lower BMI and lower baseline percent activity impairment. Furthermore, features associated with asthma control, asthma duration and medication history were common in the list of the top 25 predictors of CSEF at Year 1 (Fig 4). SHAP analysis was performed to assess the directionality of predictors of CSEF at Year 1. Overall, a lower pre-exposure rate of CSE and a residence outside of the UK were predictive of being CSEF at Year 1. Furthermore, lower baseline ACQ-5 score, BMI and baseline percent activity impairment were also associated with being CSEF at Year 1 (Fig 4). Additionally, a lower asthma duration was also associated with being CSEF at Year 1. A SHAP waterfall plot for a randomly selected patient is included in S1 Fig.

**Fig 4:**
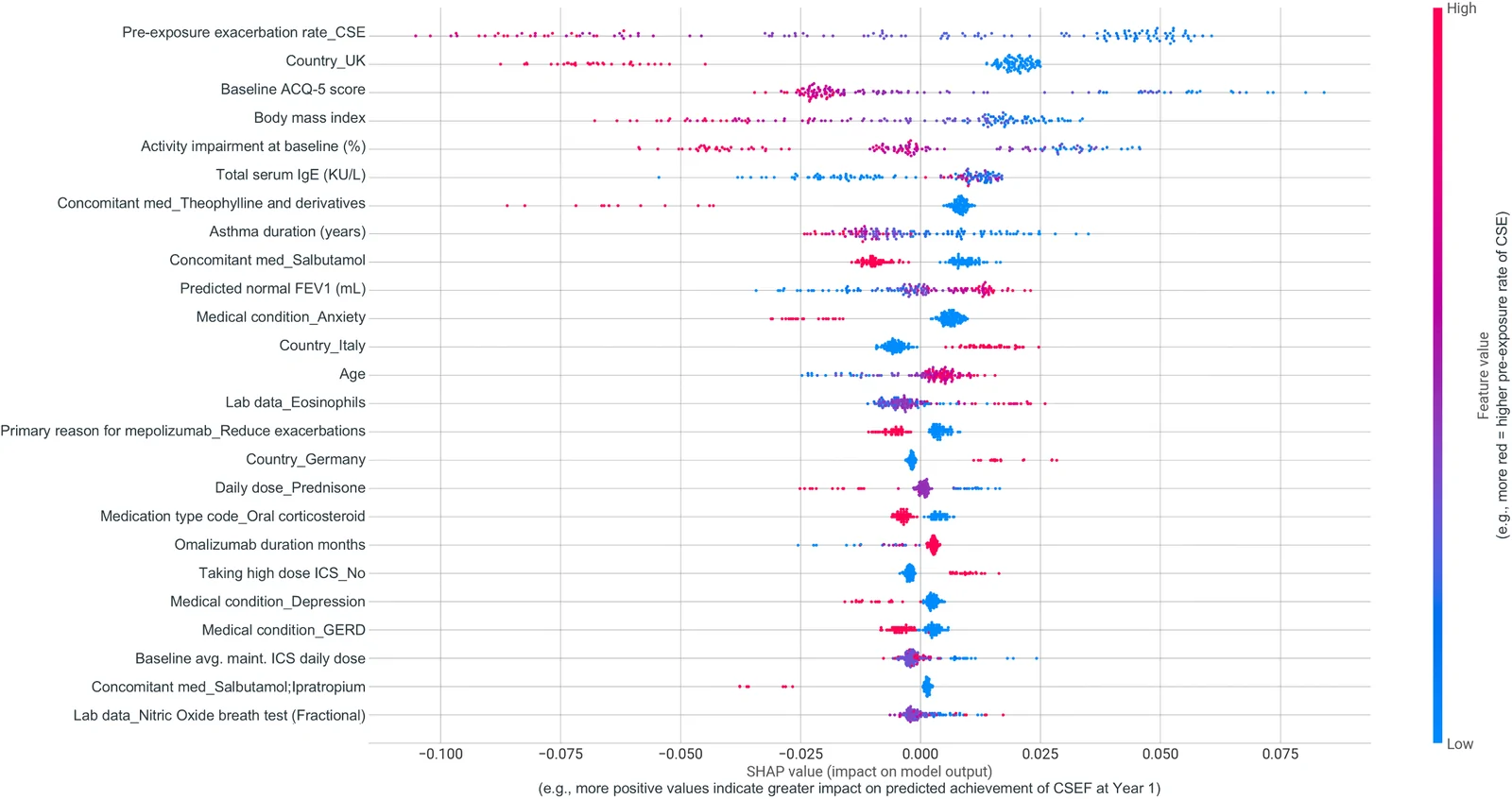
SHAP summary plot for the secondary analysis. ACQ-5, Asthma Control Questionnaire-5; BMI, body mass index; CSE, clinically significant exacerbation; FEV1, forced expiratory volume in 1 second; GERD, gastroesophageal reflux disease; ICS, inhaled corticosteroids; IgE, immunoglobulin E; OCS, oral corticosteroids; QoL, quality of life; SHAP, SHapley Additive exPlanations; UK, United Kingdom.

### Exploratory analysis: Maintained response

For analysis of maintained response, Logistic Regression, Random Forest and XGBoost had an accuracy of 64.2%, 70.8% and 72.6%, respectively. XGBoost was the best-performing model, with an accuracy of 68% and an AUC of 0.73 following cross-validation (Table 2).

Feature selection with XGBoost with SHAP post-hoc analysis was performed. The results indicated that residency outside of the UK was a top predictor of maintained response followed by lower pre-exposure rate of CSE, lower baseline ACQ-5 score, eosinophil levels, lower BMI, and higher FEV1. These results are consistent with the secondary analysis, where similar features were identified as top predictors of CSEF at Year 1 (Fig 5). A SHAP waterfall plot for a randomly selected patient included in this analysis is shown in S2 Fig.

**Fig 5:**
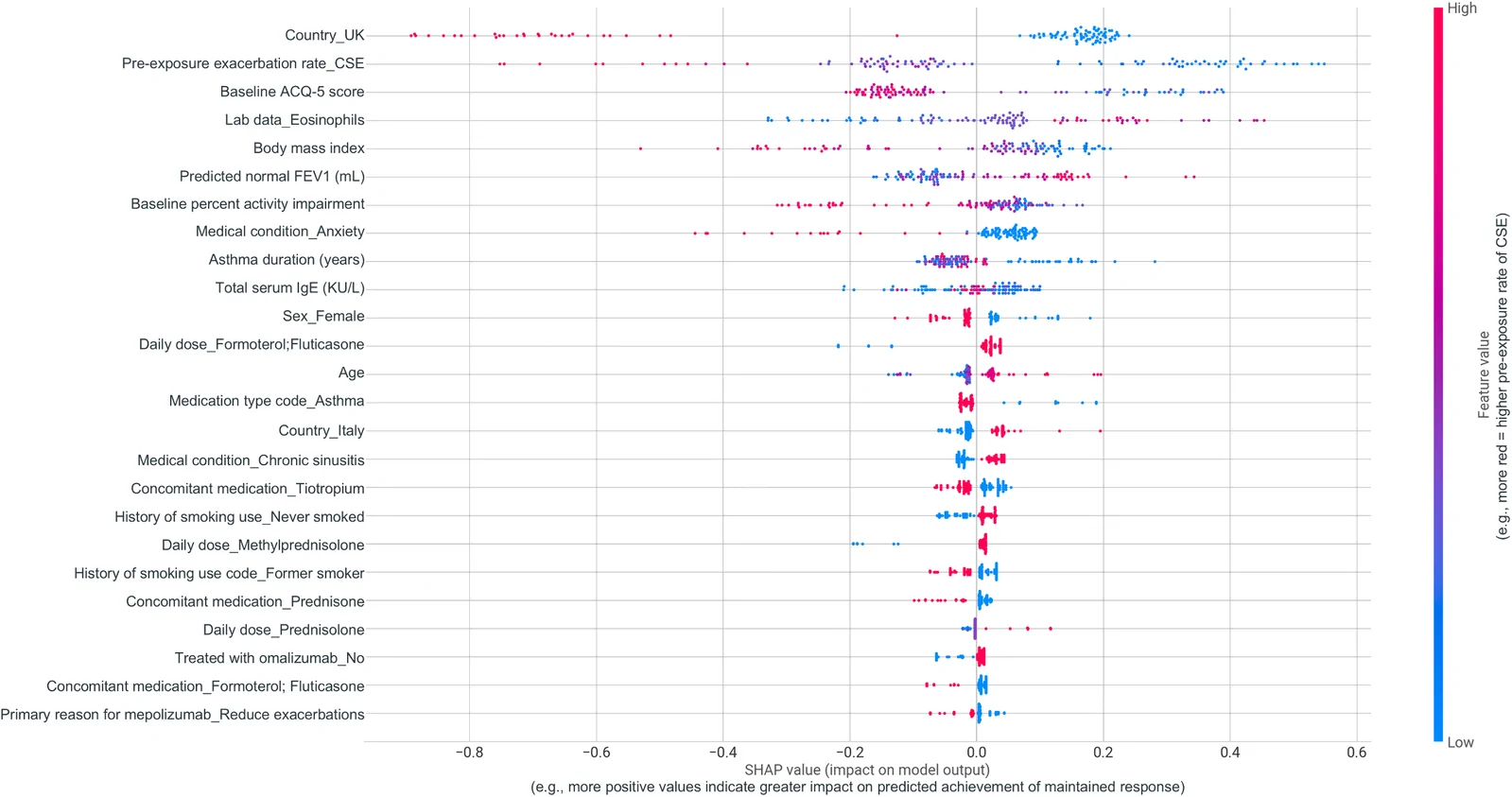
SHAP summary plot for the maintained response analysis. ACQ-5, Asthma Control Questionnaire-5; CSE, clinically significant exacerbations; FEV1, forced expiratory volume in 1 second; IgE, immunoglobulin E; SHAP, SHapley Additive exPlanations; UK, United Kingdom.

## Discussion

Utilizing data from the REALITI-A study and ML methodologies, the present study provides important clinical insights into the factors that predict mepolizumab response for severe asthma. Features consistently identified as top predictors of response were mostly those related to asthma severity. These included pre-exposure CSE rates, baseline ACQ-5 score, activity impairment at baseline, blood eosinophil levels, and total serum IgE at baseline. Qualitatively, the SHAP analysis suggests that less severe asthma, as indicated by lower activity impairment at baseline and lower ACQ-5 score at baseline, predicts greater reductions in CSE and being CSEF at Year 1 and Year 2 post-exposure. This interpretation is consistent with a previous analysis showing that patients with lower ACQ-5 scores responded better to mepolizumab [22]. This suggests that patients with lower severity will achieve and maintain control faster following treatment with mepolizumab.

Results from the primary analysis suggest that a higher rate of pre-exposure CSE predicts a greater reduction in CSE, which may in fact reflect how the primary outcome was operationalized. Specifically, patients with a higher number during pre-exposure rate of CSE have greater potential for a reduction in CSE following exposure to mepolizumab as compared to those with relatively few CSE in the pre-exposure period. Nonetheless, the observation that individuals with higher numbers of pre-exposure exacerbations were associated with greater mepolizumab response is in line with a previous study (DREAM trial), which identified a subgroup of patients with repeated exacerbations as being well-suited for mepolizumab treatment, emphasizing that the primary benefit of mepolizumab is to significantly reduce the number of asthma exacerbations [14].

Results from the present study suggest that those with a higher BMI were less likely to respond to mepolizumab at both Year 1 and Year 2 post-exposure. Previous research has also shown an association between elevated BMI and poor asthma control, perhaps partly attributable to increased usage of OCS leading to weight gain [23–26]. A recent study by Eger et al. showed, in Dutch patients with severe asthma, that those who were non-responders to anti-IL-5 biologics after 2 years of follow-up had higher BMI as compared to cohorts that were super responders [27]. Collectively, these findings are consistent with this analysis and other analyses of the REALITI-A study, which demonstrated that patients with a higher BMI had lower response to mepolizumab at Year 1 and Year 2 post-exposure [28].

The results from this study indicate that those with a lower pre-exposure rate of CSEs, ACQ-5 scores, activity impairment and BMI, and thus less severe asthma may respond faster to mepolizumab by achieving CSEF and maintaining the benefit over time. These findings can inform decision-making for healthcare professionals (HCP) and aid in identifying potential responders for mepolizumab and considering its initiation, allowing HCPs to guide patients more efficiently and offer a practical foundation for more personalized treatment strategies, thereby improving outcomes and enabling more efficient use of healthcare resources.

The machine learning models predicted that total serum IgE was a top predictor of response to mepolizumab, however, the SHAP analysis indicated that the directionality of this feature appeared to be inconclusive in all three analyses. Interestingly, a post-hoc analysis of the MENSA and MUSCA randomized clinical trials demonstrated the efficacy of mepolizumab regardless of the IgE level prior to initiating treatment [29]. This indicates that while total serum IgE level may be a top predictor of response, the ambiguity in directionality suggests that it may not be an ideal factor in guiding clinical decision-making in isolation.

The analysis in the current study also revealed that patients residing outside the UK had better responses. This may be a result of the unique clinical practice, healthcare access, or asthma severity at the time of treatment initiation in the UK. More specifically, the UK had the most restrictive reimbursement criteria among all of the countries that were included in the REALITI-A study, which required patients to have experienced a minimum of four CSEs, whereas the other countries only required two CSEs. Therefore, patients who were eligible for reimbursement for mepolizumab in the UK likely had a higher number of CSEs at baseline and perhaps more severe asthma, making it more challenging for these patients to achieve CSEF at Year 1 post-exposure and maintain response. Ultimately, geographic variability is an important consideration for clinicians, particularly when applying these findings to global populations [15,28,30]. Future research could further explore the influence of healthcare system-level factors on model performance and generalizability, for example through sensitivity analyses excluding geographic variables or through subgroup evaluations across regions, to better distinguish the impact of clinical characteristics from structural differences in treatment access.

One of the key strengths of this study is its use of tree-based models, which can identify complex, non-linear relationships between a large number of features [31,32], whereas traditional regression models may not be able to account for the complex interactions between a large set of features [33,34]. The efficiency with which ML can identify relationships between clinical variables and contribute to higher efficiency and accuracy of medical diagnosis, drug discovery and prediction of patient outcomes has been demonstrated in previous studies. For instance, in cardiovascular disease prediction, ML algorithms were able to detect subtle risk factors and interactions within patient data, leading to more accurate predictions of cardiovascular events [35]. Furthermore, the use of ML in disease diagnosis and drug discovery has demonstrated its ability to uncover complex relationships between variables, improving diagnostic accuracy and identifying effective treatments [36]. These studies collectively highlight the transformative potential of ML in healthcare, offering faster, more precise diagnostic capabilities that can significantly improve clinical decision-making and patient outcomes.

This study does have limitations that should be noted. As a secondary analysis of the REALITI-A study, it shares the same constraints of real-world observational data, including variability in data collection and potential biases. While real-world data improve the generalizability of the findings, they also introduce a level of complexity not present in controlled clinical trials. Moreover, while the ML models used in this analysis provide valuable insights, they are complex and may not always be fully interpretable by clinicians. The SHAP analysis was used to enhance interpretability, but the relationships between predictors and outcomes were described qualitatively, without quantifying the degree to which specific factors influence treatment response. This limitation highlights the need for further refinement of these models to increase their practical applicability in clinical settings.

## Conclusion

This study demonstrates the utility of ML algorithms in identifying and ranking predictors of mepolizumab response in severe asthma. While some findings align with existing literature, the use of ML has allowed for the discovery of complex, non-linear relationships that traditional methods may have overlooked. Notably, although several predictors identified in this analysis, such as pre-exposure CSE frequency, are already incorporated into current clinical decision-making frameworks, the application of ML methods enables the identification and integration of less well-established predictors and their interactions. The use of SHAP-based interpretability provided transparent identification and ranking of key drivers of response, with clear insights into the relative importance and, where applicable, the directionality of individual predictors. Importantly, the consistency of core features, including pre-exposure CSE frequency, baseline ACQ-5 score, BMI, activity impairment, and blood eosinophil counts across multiple endpoints supports the robustness of these findings. From a clinical perspective, the primary value of this approach lies in its ability to differentiate between patients who already meet treatment criteria. Even among individuals with similar exacerbation histories, the model integrates additional baseline characteristics to highlight meaningful differences in the likelihood of response. This more nuanced risk stratification may help inform treatment expectations and support more individualized management decisions in real-world practice. Overall, insights from this study pave the way for the development of predictive models that can inform and expedite more precise clinical decision-making, ultimately improving patient outcomes in the real-world management of severe asthma.

## Supporting information

S1 Table Initial set of features considered for prediction models. (DOCX)

S2 Table List of comorbidities included as variables under the Medical Condition feature. (DOCX)

S3 Table List of medications included as variables under the Drug Name Taken During Baseline Period with GSK Drug Synonym feature. (DOCX)

S4 Table Clinical characteristics of patients for each primary, secondary and exploratory analysis. (DOCX)

S1 Fig SHAP waterfall plot showing the output of the SHAP analysis for one randomly selected patient included in the secondary analysis. (TIF)

S2 Fig SHAP waterfall plot showing the output of the SHAP analysis for one randomly selected patient included in the exploratory analysis. (TIF)

## Abbreviations

ACQ-5: Asthma Control Questionnaire-5
AUC: Area Under the Curve
BMI: Body Mass Index
CSE: Clinically Significant Exacerbations
CSEF: Free from Clinically Significant Exacerbations
GERD: gastroesophageal reflux disease
GINA: Global Initiative for Asthma
HCP: Healthcare Professional
ICS: Inhaled Corticosteroids
IgE: Immunoglobulin E
kNN: k-Nearest Neighbors
LASSO: Least Absolute Shrinkage and Selection Operator
ML: Machine Learning
MAE: mean absolute error
MSE: mean squared error
OCS: Oral Corticosteroids
RMSE: root mean squared error
SHAP: SHapley Additive exPlanations
SCS: Systemic Corticosteroids
QoL: Quality of Life
10^9^ cells per liter: Grams per Liter
SD: Standard Deviation
Q1: First Quartile
Q3: Third Quartile
FEV1: Forced Expiratory Volume in 1 Second
UK: United Kingdom
XGBoost: Extreme Gradient Boosting
