# Supplementary material for: Machine learning approaches for the identification of real-world predictors of mepolizumab response
Source: PLoS One. 2026 Nov 13;21(11):e0362402. doi: 10.1371/journal.pone.0362402 (PMC13766294; doi:10.1371/journal.pone.0362402)
Supplement: Supplementary file 1 [file pone.0362402.s001.docx]

**S1** **Table**. Initial set of features considered for prediction models.

| **Label** | **Variable source** | **Dataset** | **Comments** |  |
| --- | --- | --- | --- | --- |
| Age in years | AGE | Demo |  |  |
| Sex | SEX | Demo |  |  |
| Race | RACECD | Demo |  |  |
| BMI | VSBMI | Demo |  |  |
| History of smoking use | SUSMHSCD | Demo |  |  |
| Baseline average maintenance OCS daily dose | BLMNMOCS | Demo |  |  |
| Baseline OCS use | BASEOCS | demo |  |  |
| Baseline average maintenance ICS daily dose | BLMNMICS | Demo |  |  |
| Baseline ACQ | BASEACQ | acq | where VISITNUM = 20 |  |
| Taking high-dose ICS | HDOSICS | asthist |  |  |
| Take additional asthma maintenance meds | ADASTHM | asthist |  |  |
| Oral corticosteroid maintenance therapy | OCMTPY | asthist |  |  |
| Primary reason treating with Nucala | RSTRTN | asthist |  |  |
| Asthma duration | ASTDUR | asthist |  |  |
| Drug name (Concomitant meds) taken during baseline period with GSK drug synonym^[1]^ | CMDRGSYN | conmeds | where BASEDOS = Y |  |
| Daily dose (Concomitant meds) taken during baseline period | DAILYDOS | conmeds | where BASEDOS = Y |  |
| Pre-exposure exacerbation rate of CSE | EXBRATPR_CSE | Demo |  |  |
| Baseline blood eosinophils | BEOSCD1 | Demo |  |  |
| Predicted normal FEV1 | FEV1PDML | pft | where BASEFL = Y |  |
| Employment status | WPAI1 | wpai | where VISITNUM = 20 |  |
| Base activity impairment | BASIMPA | wpai | where VISITNUM = 20 |  |
| Allergen test result at treatment index | ALGRES | algtst | where VISITNUM = 20 |  |
| Laboratory test performed | LBTEST | lab | where VISITNUM = 20 |  |
| Laboratory test result | LBSTRESN | lab | where VISITNUM = 20 |  |
| Unscheduled asthma-related healthcare contacts at treatment index | UNSCCONT | unshcc | where VISITNUM = 20 |  |
| Country | COUNTRY | Demo |  |  |
| Medication type code (Concomitant meds) taken during baseline period | CMTYPCD | conmeds | where BASEDOS = Y |  |
| Unit dose (Concomitant meds) taken during baseline period | CMUDOS | conmeds | where BASEDOS = Y |  |
| Frequency (Concomitant meds) taken during baseline period | CMFREQ | conmeds | where BASEDOS = Y |  |
| Treated with Xolair | TRTXOL | asthist |  |  |
| Xolair duration months | XODURMO | asthist |  |  |
| Medical condition^[2]^ | MHTERM | medhist |  | |

ACQ: Asthma Control Questionnaire; CSE: Clinically significant exacerbation; OCS: oral corticosteroid; BMI: Body mass index; FEV1: Forced expiratory volume in the first second

Note: all these variables are collected during the Pre-exposure period, thus they are available at baseline

For variables taken at VISITNUM = 20, in case of missing value, the most recent value with VISITNUM < 20 will be considered

[1] On the basis of population prevalence and clinical importance, 29 concomitant drugs will be included as a separate binary feature, each one having its own ranking score in the final results

[2] Only the 23 most common comorbidities among the eligible population will be considered and included as a separate binary feature, each one having its own ranking score in the final results
