# Supplementary material for: Machine learning approaches for the identification of real-world predictors of mepolizumab response
Source: PLoS One. 2026 Nov 13;21(11):e0362402. doi: 10.1371/journal.pone.0362402 (PMC13766294; doi:10.1371/journal.pone.0362402)
Supplement: Supplementary file 2 [file pone.0362402.s002.docx]

**S2 Table.** List of comorbidities included as variables under the Medical Condition feature.

| **Class** | **Comorbidity** |
| --- | --- |
| Asthma disorders | Hay fever |
| Asthma disorders | Chronic Sinusitis |
| Asthma disorders | Nasal polyp |
| Other comorbid disorders | Gastroesophageal reflux disease |
| Asthma disorders | Drug hypersensitivity |
| Asthma disorders | Nasal polypectomy |
| Other comorbid disorders | Hyperlipidemia |
| Other comorbid disorders | Anxiety |
| Other comorbid disorders | Depression |
| Other comorbid disorders | Osteoporosis |
| Other comorbid disorders | Lung disease |
| Other comorbid disorders | Broken bones |
| Other comorbid disorders | Diabetes |
| Other comorbid disorders | Food allergy |
| Asthma disorders | Atopic dermatitis |
| Other comorbid disorders | Cataract |
| Other comorbid disorders | Chronic obstructive pulmonary disease |
| Other comorbid disorders | Pneumonia |
| Asthma disorders | Anaphylaxis |
| Other comorbid disorders | Oropharyngeal candidiasis |
| Other comorbid disorders | Churg Strauss syndrome |
| Other comorbid disorders | Hyper eosinophilic syndrome |
| Other comorbid disorders | Vasculitis |
