# Supplementary material for: Machine learning approaches for the identification of real-world predictors of mepolizumab response
Source: PLoS One. 2026 Nov 13;21(11):e0362402. doi: 10.1371/journal.pone.0362402 (PMC13766294; doi:10.1371/journal.pone.0362402)
Supplement: Supplementary file 3 [file pone.0362402.s003.docx]

**S3 Table**. List of medications included as variables under the Drug Name Taken During Baseline Period with GSK Drug Synonym feature.

**Medication**

Beclometasone dipropionate;formoterol fumarate

Budesonide;formoterol fumarate

Fluticasone furoate;vilanterol trifenatate

Salbutamol

Salmeterol xinafoate;fluticasone propionate

Tiotropium bromide

Prednisolone

Prednisone

Formoterol fumarate;fluticasone propionate

Montelukast

Methylprednisolone

Theophylline

Mometasone furoate;formoterol fumarate

Salbutamol sulfate;ipratropium bromide

Fenoterol hydrobromide;ipratropium bromide

Fluticasone propionate

Fluticasone

Budesonide

Aclidinium bromide

CICLESONIDE or IPRATROPIUM

Ipratropium bromide

Umeclidinium bromide

Beclometasone dipropionate

Formoterol

Carbocisteine

Salmeterol

Glycopyrronium bromide

Rupatadine

Terbutaline sulfate
