# Supplementary material for: Machine learning approaches for the identification of real-world predictors of mepolizumab response
Source: PLoS One. 2026 Nov 13;21(11):e0362402. doi: 10.1371/journal.pone.0362402 (PMC13766294; doi:10.1371/journal.pone.0362402)
Supplement: Supplementary file 4 [file pone.0362402.s004.docx]

**S4 Table.** Clinical characteristics of patients for each primary, secondary and exploratory analysis.

| **Clinical characteristics** | **Primary and secondary analyses  (N = 685)** | **Exploratory analysis (N = 529)** |
| --- | --- | --- |
| **Exacerbations requiring an ED visit and/or hospitalization** | | |
| n | 685 | 529 |
| Mean (SD) | 0.85 (1.83) | 0.74 (1.55) |
| Median (min, max) | 0 (0, 17) | 0 (0, 10) |
| Q1, Q3 | 0, 1 | 0, 1 |
| 0, n (%) | 456 (66.57) | 365 (69.00) |
| 1, n (%) | 109 (15.91) | 83 (15.69) |
| 2, n (%) | 45 (6.57) | 31 (5.86) |
| 3, n (%) | 24 (3.50) | 15 (2.84) |
| 4, n (%) | 14 (2.04) | 8 (1.51) |
| 5, n (%) | 15 (2.19) | 12 (2.27) |
| >5, n (%) | 22 (3.21) | 15 (2.84) |
| **Exacerbations requiring hospitalization** | | |
| n | 685 | 529 |
| Mean (SD) | 0.41 (1.05) | 0.33 (0.81) |
| Median (min, max) | 0 (0, 10) | 0 (0, 5) |
| Q1, Q3 | 0, 0 | 0, 0 |
| 0, n (%) | 531 (77.52) | 421 (79.58) |
| 1, n (%) | 95 (13.87) | 72 (13.61) |
| 2, n (%) | 29 (4.23) | 19 (3.59) |
| 3, n (%) | 15 (2.19) | 9 (1.7) |
| 4, n (%) | 4 (0.58) | 3 (0.57) |
| 5, n (%) | 7 (1.02) | 5 (0.95) |
| >5, n (%) | 4 (0.58) | 0 (0.00) |
| **Pre-bronchodilator FEV1 (mL) ^a^** | | |
| n (%) | 349 (50.95) | 277 (52.36) |
| Mean (SD) | 2007.26 (815.35) | 2005.11 (778.72) |
| Median (min, max) | 1930 (470, 4860) | 1970 (470, 4860) |
| Q1, Q3 | 1410, 2520 | 1420, 2500 |
| **Pre-bronchodilator FVC (mL) ^a^** | | |
| n (%) | 348 (50.80) | 276 (52.17) |
| Mean (SD) | 2994.29 (1039.82) | 2985.77 (996.63) |
| Median (min, max) | 2885 (780, 6820) | 2870 (780, 6070) |
| Q1, Q3 | 2257.5, 3652.5 | 2287.5, 3652.5 |
| **Pre-bronchodilator FEV1/FVC ^a^** | | |
| n (%) | 348 (50.80) | 276 (52.17) |
| Mean (SD) | 0.67 (0.13) | 0.67 (0.13) |
| Median (min, max) | 0.67 (0.31, 1) | 0.67 (0.31, 1) |
| Q1, Q3 | 0.58, 0.75 | 0.58, 0.76 |
| **Pre-bronchodilator percent predicted FEV1 (%) ^a^** | | |
| n (%) | 349 (50.95) | 277 (52.36) |
| Mean (SD) | 67.92 (20.71) | 68.44 (20.09) |
| Median (min, max) | 68.54 (19.11, 132.88) | 69.35 (22.08, 132.88) |
| Q1, Q3 | 52.15, 82.47 | 54.12, 82.35 |
| **Reversibility (%)** | | |
| n (%) | 68 (9.93) | 57 (10.78) |
| Mean (SD) | 7.92 (8.74) | 7.34 (8.17) |
| Median (min, max) | 6.5 (-12.79, 36) | 6.33 (-12.79, 28.87) |
| Q1, Q3 | 0.91, 13.48 | 0.92, 11.86 |
| **Post-bronchodilator FEV1 (mL)** | | |
| n (%) | 124 (18.10) | 102 (19.28) |
| Mean (SD) | 2148.08 (911.38) | 2171.39 (888.87) |
| Median (min, max) | 2100 (500, 5000) | 2120 (500, 5000) |
| Q1, Q3 | 1442.5, 2680 | 1455, 2637.5 |

Note: Baseline is the value at index date or the most recent value available prior to index date, but not prior to 90 days before index date.

Results include exacerbations recorded during the 12-month pre-enrollment period, plus any exacerbations with a start date during the run-in the period, where run-in is defined as the period between enrollment and index dates inclusively.

If a measure of zero is reported, it is imputed with half of the lowest possible measurement prior to log transforming the data used in the geometric mean and SD log calculations.

*n the ‘Any’ categories, participants were counted only once. Those with any events were counted under ‘Yes’ while those with no events for all relevant categories were counted under ‘No’. The other categories are not mutually exclusive, wherein patients may be captured under multiple categories if they had multiple medical conditions of interest.

=

a. Pre-bronchodilator assessments were lung function assessments performed at the clinic and participants could have taken their daily bronchodilator therapy prior to assessment. There was no formal requirement to withhold short- or long-acting bronchodilators.

GI/L (grams per liter), SD (standard deviation), min (minimum), max (maximum), Q1 (first quartile), Q3 (third quartile), ED (emergency department), ACQ-5 (Asthma Control Questionnaire - 5 items), FEV1 (forced expiratory volume in 1 second), FVC (forced vital capacity),
