# Supplementary material for: Machine learning approaches for the identification of real-world predictors of mepolizumab response
Source: PLoS One. 2026 Nov 13;21(11):e0362402. doi: 10.1371/journal.pone.0362402 (PMC13766294; doi:10.1371/journal.pone.0362402)
Supplement: Supplementary file 5 [file pone.0362402.s007.docx]

REALITI-A previously demonstrated the real-world effectiveness of mepolizumab in patients with severe asthma. Building on this dataset, the current study applies machine learning approaches to explore heterogeneity in treatment response and to identify potential clinical predictors associated with differential treatment outcomes, including exacerbation reduction, achievement of CSE-free status, and maintenance of response over time.

Overall, the manuscript is well written, and the use of three complementary analyses to assess treatment response is a strength of the study. However, I have a few suggestions that may help further improve the methodological transparency and interpretability of the results.

1. The best-performing model differed across the outcome analyses (e.g., XGBoost for the primary outcome). While this may reflect differences in outcome structure and signal patterns, additional details on the model development workflow would help readers better understand the robustness of model selection. Specifically, it would be helpful to clarify how the dataset was partitioned into training and hold-out sets, how cross-validation was implemented, and how preprocessing and feature selection were incorporated within this pipeline. Because different models were selected across the three outcome analyses, please report the modeling workflow separately for each analysis.

2. The identification of geographic location (e.g., residence outside of the UK) as a top predictive feature is interesting. The authors appropriately acknowledge that this likely reflects healthcare system differences (e.g., reimbursement criteria and treatment initiation thresholds) rather than underlying biological differences. However, given that this feature ranks highly in the predictive model, it raises questions regarding the transportability and generalizability of the model across healthcare systems with different treatment access patterns. To better understand whether this signal reflects underlying disease biology versus healthcare system structure, the authors may consider performing sensitivity analyses, such as retraining the model excluding geographic variables, or evaluating model performance separately within UK and non-UK subgroups. Such analyses would help clarify whether model performance is driven primarily by clinical predictors versus system-level factors and would strengthen confidence in the model’s generalizability.

3. The conclusion section appears somewhat descriptive and could be more specific in terms of the clinical implications of the findings. Currently, mepolizumab is prescribed primarily for patients with severe asthma, and in clinical practice, pre-exposure CSE frequency is already an important component of treatment decision-making. In this context, it would be helpful if the authors could more clearly articulate how the proposed modeling approach provides additional value beyond existing clinical decision frameworks. In particular, the authors may consider clarifying how confident they are that these findings could support a more personalized medicine approach in real-world clinical practice, and to what extent the model could meaningfully influence treatment selection beyond current severity-based prescribing criteria. While strengthening the clinical interpretation would enhance the impact of the manuscript, I would like to clarify that I believe the scientific value of the study is not dependent on whether the model ultimately proves to be clinically actionable.
