# Supplementary material for: Machine learning approaches for the identification of real-world predictors of mepolizumab response
Source: PLoS One. 2026 Nov 13;21(11):e0362402. doi: 10.1371/journal.pone.0362402 (PMC13766294; doi:10.1371/journal.pone.0362402)
Supplement: Supplementary file 6 [file pone.0362402.s009.docx]

**Responses to Reviewer Comments**

Reviewer #1: REALITI-A previously demonstrated the real-world effectiveness of mepolizumab in patients with severe asthma. Building on this dataset, the current study applies machine learning approaches to explore heterogeneity in treatment response and to identify potential clinical predictors associated with differential treatment outcomes, including exacerbation reduction, achievement of CSE-free status, and maintenance of response over time.

Overall, the manuscript is well written, and the use of three complementary analyses to assess treatment response is a strength of the study. However, I have a few suggestions that may help further improve the methodological transparency and interpretability of the results.

1. The best-performing model differed across the outcome analyses (e.g., XGBoost for the primary outcome). While this may reflect differences in outcome structure and signal patterns, additional details on the model development workflow would help readers better understand the robustness of model selection. Specifically, it would be helpful to clarify how the dataset was partitioned into training and hold-out sets, how cross-validation was implemented, and how preprocessing and feature selection were incorporated within this pipeline. Because different models were selected across the three outcome analyses, please report the modeling workflow separately for each analysis.

**Response:** Thank you for this comment. We would like to note that although different models were selected, the same modeling workflow was applied to each of the three outcome analyses. For each study endpoint, prediction algorithms were built and compared using the following steps: data preparation, training and evaluation of the model, and final interpretation of feature rankings. These details have been more clearly articulated in the Machine learning techniques section of the Methodology (Lines 171-202).

2. The identification of geographic location (e.g., residence outside of the UK) as a top predictive feature is interesting. The authors appropriately acknowledge that this likely reflects healthcare system differences (e.g., reimbursement criteria and treatment initiation thresholds) rather than underlying biological differences. However, given that this feature ranks highly in the predictive model, it raises questions regarding the transportability and generalizability of the model across healthcare systems with different treatment access patterns. To better understand whether this signal reflects underlying disease biology versus healthcare system structure, the authors may consider performing sensitivity analyses, such as retraining the model excluding geographic variables, or evaluating model performance separately within UK and non-UK subgroups. Such analyses would help clarify whether model performance is driven primarily by clinical predictors versus system-level factors and would strengthen confidence in the model’s generalizability.

**Response:** Thank you for these thoughtful insights. We agree that these are important considerations for model transportability; however, these analyses are not in scope with the current study. Therefore, we have included these considerations as directions for future research in the Discussion:

Lines 368-372: Future research could further explore the influence of healthcare system-level factors on model performance and generalizability, for example through sensitivity analyses excluding geographic variables or through subgroup evaluations across regions, to better distinguish the impact of clinical characteristics from structural differences in treatment access.

3. The conclusion section appears somewhat descriptive and could be more specific in terms of the clinical implications of the findings. Currently, mepolizumab is prescribed primarily for patients with severe asthma, and in clinical practice, pre-exposure CSE frequency is already an important component of treatment decision-making. In this context, it would be helpful if the authors could more clearly articulate how the proposed modeling approach provides additional value beyond existing clinical decision frameworks. In particular, the authors may consider clarifying how confident they are that these findings could support a more personalized medicine approach in real-world clinical practice, and to what extent the model could meaningfully influence treatment selection beyond current severity-based prescribing criteria. While strengthening the clinical interpretation would enhance the impact of the manuscript, I would like to clarify that I believe the scientific value of the study is not dependent on whether the model ultimately proves to be clinically actionable.

**Response:** Thank you for this comment. The following has been added to strengthen our Conclusion:

Lines 404-418: Notably, although several predictors identified in this analysis, such as pre-exposure CSE frequency, are already incorporated into current clinical decision-making frameworks, the application of ML methods enables the identification and integration of less well-established predictors and their interactions. The use of SHAP-based interpretability provided transparent identification and ranking of key drivers of response, with clear insights into the relative importance and, where applicable, the directionality of individual predictors. Importantly, the consistency of core features, including pre-exposure CSE frequency, baseline ACQ-5 score, BMI, activity impairment, and blood eosinophil counts across multiple endpoints supports the robustness of these findings. From a clinical perspective, the primary value of this approach lies in its ability to differentiate between patients who already meet treatment criteria. Even among individuals with similar exacerbation histories, the model integrates additional baseline characteristics to highlight meaningful differences in the likelihood of response. This more nuanced risk stratification may help inform treatment expectations and support more individualized management decisions in real-world practice. Overall, insights from this study pave the way for the development of predictive models that can inform and expedite more precise clinical decision-making, ultimately improving patient outcomes in the real-world management of severe asthma.

Reviewer #2: The manuscript is good and worse publishing

The article presents a robust application of machine learning (ML) methodologies to identify real-world predictors of mepolizumab response in patients with severe asthma using the REALITI-A dataset. The study is well-designed, utilizing advanced interpretability tools like SHAP to bridge the gap between complex model outputs and clinical utility.

Typographical Errors:

The manuscript is generally written in standard English and is intelligible. However, several minor errors and inconsistencies should be addressed to meet the journal's standard for clear and correct language. Here are some examples: On page 2, GSK plc is incorrectly written as GSK pic. On page 7, the data availability section contains a placeholder error: replace any insta instances of........

**Response:** Thank you for pointing these out. These errors have been corrected.

Data Availability:

The authors note that data access is restricted and available upon request. Given the journal's policy, the authors should ensure this level of restriction is fully justified by legal or ethical constraints.

**Response:** Our Data availability statement has been updated as follows: Please refer to GSK weblink to access GSK’s data sharing policies and as applicable seek anonymized subject level data via the link https://www.gsk-studyregister.com/, for this study (204710). This article reports secondary analyses using those existing anonymized data and does not generate any new patient-level datasets.

Conflict Disclosure:

The authors have clearly disclosed that several researchers are employees and shareholders of GSK, the manufacturer of mepolizumab. This transparency is essential given the potential for perceived bias.

**Response:** We have updated our Competing Interests Statement and Funding Statement to meet the journal’s requirements of transparency.

Competing Interests Statement: This study was funded by GSK (215038). Rafael Alfonso-Cristancho, Lingjiao Zhang, Peter Howarth and Koyo Usuba are employees and shareholders of GSK. Xinyang Liu, Tim Tian Yu Han, Natalie Nightingale and Ali Tehrani are employees of IQVIA. This commercial affiliation does not alter our adherence to PLOS ONE policies on sharing data and materials.

Funding Statement: This research was supported by GSK plc through grant number 215038. The funding agency provided support in the form of salaries for authors Rafael Alfonso-Cristancho, Lingjiao Zhang, Peter Howarth and Koyo Usuba, who are employees and shareholders of GSK plc. GSK plc paid consulting fees to IQVIA for study design, data analysis, and manuscript development services. Authors Xinyang Liu, Tim Tian Yu Han, Natalie Nightingale and Ali Tehrani are employees of IQVIA. The specific roles of the authors are articulated in the ‘author contributions’ section. The funder provided support in the form of salaries for authors (RA, LZ, PH, and KU), but had no additional role in study design, data collection and analysis, decision to publish, or preparation of the manuscript beyond the employment of the authors and the provision of the grant and contracting of IQVIA consulting services. No additional external funding was received for this study.
