# Supplementary material for: Machine learning approaches for the identification of real-world predictors of mepolizumab response
Source: PLoS One. 2026 Nov 13;21(11):e0362402. doi: 10.1371/journal.pone.0362402 (PMC13766294; doi:10.1371/journal.pone.0362402)
Supplement: Peer review history

# Peer review: Decision Letter 0

**DOI:** 10.1371/journal.pone.0362402.r001

**Authors:** Kwang-Sig Lee

26 Feb 2026

Dear Dr. Alfonso-Cristancho,

Thank you for submitting your manuscript to PLOS ONE. After careful consideration, we feel that it has merit but does not fully meet PLOS ONE’s publication criteria as it currently stands. Therefore, we invite you to submit a revised version of the manuscript that addresses the points raised during the review process.

Please submit your revised manuscript by Apr 12 2026 11:59PM. If you will need more time than this to complete your revisions, please reply to this message or contact the journal office at plosone@plos.org. When you're ready to submit your revision, log on to https://www.editorialmanager.com/pone/ and select the 'Submissions Needing Revision' folder to locate your manuscript file.

- A letter that responds to each point raised by the academic editor and reviewer(s). You should upload this letter as a separate file labeled 'Response to Reviewers'.
- A marked-up copy of your manuscript that highlights changes made to the original version. You should upload this as a separate file labeled 'Revised Manuscript with Track Changes'.
- An unmarked version of your revised paper without tracked changes. You should upload this as a separate file labeled 'Manuscript'.

If you would like to make changes to your financial disclosure, please include your updated statement in your cover letter. Guidelines for resubmitting your figure files are available below the reviewer comments at the end of this letter.

If applicable, we recommend that you deposit your laboratory protocols in protocols.io to enhance the reproducibility of your results. Protocols.io assigns your protocol its own identifier (DOI) so that it can be cited independently in the future. For instructions see: https://journals.plos.org/plosone/s/submission-guidelines#loc-laboratory-protocols. Additionally, PLOS ONE offers an option for publishing peer-reviewed Lab Protocol articles, which describe protocols hosted on protocols.io. Read more information on sharing protocols at https://plos.org/protocols?utm_medium=editorial-email&utm_source=authorletters&utm_campaign=protocols.

We look forward to receiving your revised manuscript.

Kind regards,

Kwang-Sig Lee

Academic Editor

PLOS One

Journal Requirements:

When submitting your revision, we need you to address these additional requirements.

1. Please ensure that your manuscript meets PLOS ONE's style requirements, including those for file naming. The PLOS ONE style templates can be found at

https://journals.plos.org/plosone/s/file?id=wjVg/PLOSOne_formatting_sample_main_body.pdf and

https://journals.plos.org/plosone/s/file?id=ba62/PLOSOne_formatting_sample_title_authors_affiliations.pdf

2. Please note that PLOS One has specific guidelines on code sharing for submissions in which author-generated code underpins the findings in the manuscript. In these cases, we expect all author-generated code to be made available without restrictions upon publication of the work. Please review our guidelines at https://journals.plos.org/plosone/s/materials-and-software-sharing#loc-sharing-code and ensure that your code is shared in a way that follows best practice and facilitates reproducibility and reuse.

3. Thank you for stating the following in the Competing Interests section: [I have read the journal's policy and the authors of this manuscript have the following competing interests: Rafael Alfonso-Cristancho, Lingjiao Zhang, Peter Howarth and Koyo Usuba are employees and shareholders of GSK. Xinyang Liu, Tim Tian Yu Han, Natalie Nightingale and Ali Tehrani are employees of IQVIA.].

Please confirm that this does not alter your adherence to all PLOS ONE policies on sharing data and materials, by including the following statement: ""This does not alter our adherence to  PLOS ONE policies on sharing data and materials.” (as detailed online in our guide for authors http://journals.plos.org/plosone/s/competing-interests).  If there are restrictions on sharing of data and/or materials, please state these. Please note that we cannot proceed with consideration of your article until this information has been declared.

Please include your updated Competing Interests statement in your cover letter; we will change the online submission form on your behalf.

4. In the online submission form, you indicated that [Requests to access to the data can be made by contacting the corresponding author.].

All PLOS journals now require all data underlying the findings described in their manuscript to be freely available to other researchers, either 1. In a public repository, 2. Within the manuscript itself, or 3. Uploaded as supplementary information.

This policy applies to all data except where public deposition would breach compliance with the protocol approved by your research ethics board. If your data cannot be made publicly available for ethical or legal reasons (e.g., public availability would compromise patient privacy), please explain your reasons on resubmission and your exemption request will be escalated for approval.

5. PLOS requires an ORCID iD for the corresponding author in Editorial Manager on papers submitted after December 6th, 2016. Please ensure that you have an ORCID iD and that it is validated in Editorial Manager. To do this, go to ‘Update my Information’ (in the upper left-hand corner of the main menu), and click on the Fetch/Validate link next to the ORCID field. This will take you to the ORCID site and allow you to create a new iD or authenticate a pre-existing iD in Editorial Manager.

6. Thank you for stating the following in the Competing Interests section: [I have read the journal's policy and the authors of this manuscript have the following competing interests: Rafael Alfonso-Cristancho, Lingjiao Zhang, Peter Howarth and Koyo Usuba are employees and shareholders of GSK. Xinyang Liu, Tim Tian Yu Han, Natalie Nightingale and Ali Tehrani are employees of IQVIA.].

We note that one or more of the authors are employed by a commercial company: GSK and IQVIA Solutions Canada Inc.

1. Please provide an amended Funding Statement declaring this commercial affiliation, as well as a statement regarding the Role of Funders in your study. If the funding organization did not play a role in the study design, data collection and analysis, decision to publish, or preparation of the manuscript and only provided financial support in the form of authors' salaries and/or research materials, please review your statements relating to the author contributions, and ensure you have specifically and accurately indicated the role(s) that these authors had in your study. You can update author roles in the Author Contributions section of the online submission form.

Please also include the following statement within your amended Funding Statement.

“The funder provided support in the form of salaries for authors [insert relevant initials], but did not have any additional role in the study design, data collection and analysis, decision to publish, or preparation of the manuscript. The specific roles of these authors are articulated in the ‘author contributions’ section.”

If your commercial affiliation did play a role in your study, please state and explain this role within your updated Funding Statement.

2. Please also provide an updated Competing Interests Statement declaring this commercial affiliation along with any other relevant declarations relating to employment, consultancy, patents, products in development, or marketed products, etc.

Within your Competing Interests Statement, please confirm that this commercial affiliation does not alter your adherence to all PLOS ONE policies on sharing data and materials by including the following statement: ""This does not alter our adherence to  PLOS ONE policies on sharing data and materials.” (as detailed online in our guide for authors http://journals.plos.org/plosone/s/competing-interests) . If this adherence statement is not accurate and  there are restrictions on sharing of data and/or materials, please state these. Please note that we cannot proceed with consideration of your article until this information has been declared.

Please include both an updated Funding Statement and Competing Interests Statement in your cover letter. We will change the online submission form on your behalf.

7. Please remove all personal information, ensure that the data shared are in accordance with participant consent, and re-upload a fully anonymized data set.

Note: spreadsheet columns with personal information must be removed and not hidden as all hidden columns will appear in the published file.

Additional guidance on preparing raw data for publication can be found in our Data Policy (https://journals.plos.org/plosone/s/data-availability#loc-human-research-participant-data-and-other-sensitive-data) and in the following article: http://www.bmj.com/content/340/bmj.c181.long.

8. If the reviewer comments include a recommendation to cite specific previously published works, please review and evaluate these publications to determine whether they are relevant and should be cited. There is no requirement to cite these works unless the editor has indicated otherwise.

[Note: HTML markup is below. Please do not edit.]

Reviewers' comments:

Reviewer's Responses to Questions

**Comments to the Author**

1. Is the manuscript technically sound, and do the data support the conclusions?

Reviewer #1: Yes

Reviewer #2: Yes

**********

2. Has the statistical analysis been performed appropriately and rigorously?

Reviewer #1: Yes

Reviewer #2: Yes

**********

3. Have the authors made all data underlying the findings in their manuscript fully available?

The PLOS Data policy

Reviewer #1: Yes

Reviewer #2: No

**********

4. Is the manuscript presented in an intelligible fashion and written in standard English?

Reviewer #1: Yes

Reviewer #2: Yes

**********

Reviewer #1: REALITI-A previously demonstrated the real-world effectiveness of mepolizumab in patients with severe asthma. Building on this dataset, the current study applies machine learning approaches to explore heterogeneity in treatment response and to identify potential clinical predictors associated with differential treatment outcomes, including exacerbation reduction, achievement of CSE-free status, and maintenance of response over time.

Overall, the manuscript is well written, and the use of three complementary analyses to assess treatment response is a strength of the study. However, I have a few suggestions that may help further improve the methodological transparency and interpretability of the results.

1. The best-performing model differed across the outcome analyses (e.g., XGBoost for the primary outcome). While this may reflect differences in outcome structure and signal patterns, additional details on the model development workflow would help readers better understand the robustness of model selection. Specifically, it would be helpful to clarify how the dataset was partitioned into training and hold-out sets, how cross-validation was implemented, and how preprocessing and feature selection were incorporated within this pipeline. Because different models were selected across the three outcome analyses, please report the modeling workflow separately for each analysis.

2. The identification of geographic location (e.g., residence outside of the UK) as a top predictive feature is interesting. The authors appropriately acknowledge that this likely reflects healthcare system differences (e.g., reimbursement criteria and treatment initiation thresholds) rather than underlying biological differences. However, given that this feature ranks highly in the predictive model, it raises questions regarding the transportability and generalizability of the model across healthcare systems with different treatment access patterns. To better understand whether this signal reflects underlying disease biology versus healthcare system structure, the authors may consider performing sensitivity analyses, such as retraining the model excluding geographic variables, or evaluating model performance separately within UK and non-UK subgroups. Such analyses would help clarify whether model performance is driven primarily by clinical predictors versus system-level factors and would strengthen confidence in the model’s generalizability.

3. The conclusion section appears somewhat descriptive and could be more specific in terms of the clinical implications of the findings. Currently, mepolizumab is prescribed primarily for patients with severe asthma, and in clinical practice, pre-exposure CSE frequency is already an important component of treatment decision-making. In this context, it would be helpful if the authors could more clearly articulate how the proposed modeling approach provides additional value beyond existing clinical decision frameworks. In particular, the authors may consider clarifying how confident they are that these findings could support a more personalized medicine approach in real-world clinical practice, and to what extent the model could meaningfully influence treatment selection beyond current severity-based prescribing criteria. While strengthening the clinical interpretation would enhance the impact of the manuscript, I would like to clarify that I believe the scientific value of the study is not dependent on whether the model ultimately proves to be clinically actionable.

Reviewer #2: The manuscript is good and worse publishing

The article presents a robust application of machine learning (ML) methodologies to identify real-world predictors of mepolizumab response in patients with severe asthma using the REALITI-A dataset. The study is well-designed, utilizing advanced interpretability tools like SHAP to bridge the gap between complex model outputs and clinical utility.

Typographical Errors:

The manuscript is generally written in standard English and is intelligible. However, several minor errors and inconsistencies should be addressed to meet the journal's standard for clear and correct language. Here are some examples: On page 2, GSK plc is incorrectly written as GSK pic. On page 7, the data availability section contains a placeholder error: replace any insta instances of........

Data Availability:

The authors note that data access is restricted and available upon request. Given the journal's policy, the authors should ensure this level of restriction is fully justified by legal or ethical constraints.

Conflict Disclosure:

The authors have clearly disclosed that several researchers are employees and shareholders of GSK, the manufacturer of mepolizumab. This transparency is essential given the potential for perceived bias.

**********

what does this mean?). If published, this will include your full peer review and any attached files.

If you choose “no”, your identity will remain anonymous but your review may still be made public.

**Do you want your identity to be public for this peer review?**  For information about this choice, including consent withdrawal, please see our Privacy Policy

Reviewer #1: **Yes:**  Hyunkoo Kang

Reviewer #2: **Yes:**  Walyeldin Elfakey, Professor of Pediatrics and Infectious Diseases, Medical Educator, University of Bahri, Sudan

**********

[NOTE: If reviewer comments were submitted as an attachment file, they will be attached to this email and accessible via the submission site. Please log into your account, locate the manuscript record, and check for the action link "View Attachments". If this link does not appear, there are no attachment files.]

To ensure your figures meet our technical requirements, please review our figure guidelines: https://journals.plos.org/plosone/s/figures

You may also use PLOS’s free figure tool, NAAS, to help you prepare publication quality figures: https://journals.plos.org/plosone/s/figures#loc-tools-for-figure-preparation.

NAAS will assess whether your figures meet our technical requirements by comparing each figure against our figure specifications.

Attachment Submitted filename: PONE-D-25-42854_REVIEW.docx

# Author comment: Author response to Decision Letter 1

**DOI:** 10.1371/journal.pone.0362402.r002

6 Aug 2026

Reviewer #1: REALITI-A previously demonstrated the real-world effectiveness of mepolizumab in patients with severe asthma. Building on this dataset, the current study applies machine learning approaches to explore heterogeneity in treatment response and to identify potential clinical predictors associated with differential treatment outcomes, including exacerbation reduction, achievement of CSE-free status, and maintenance of response over time.

Overall, the manuscript is well written, and the use of three complementary analyses to assess treatment response is a strength of the study. However, I have a few suggestions that may help further improve the methodological transparency and interpretability of the results.

1. The best-performing model differed across the outcome analyses (e.g., XGBoost for the primary outcome). While this may reflect differences in outcome structure and signal patterns, additional details on the model development workflow would help readers better understand the robustness of model selection. Specifically, it would be helpful to clarify how the dataset was partitioned into training and hold-out sets, how cross-validation was implemented, and how preprocessing and feature selection were incorporated within this pipeline. Because different models were selected across the three outcome analyses, please report the modeling workflow separately for each analysis.

Response: Thank you for this comment. We would like to note that although different models were selected, the same modeling workflow was applied to each of the three outcome analyses. For each study endpoint, prediction algorithms were built and compared using the following steps: data preparation, training and evaluation of the model, and final interpretation of feature rankings. These details have been more clearly articulated in the Machine learning techniques section of the Methodology (Lines 171-202).

2. The identification of geographic location (e.g., residence outside of the UK) as a top predictive feature is interesting. The authors appropriately acknowledge that this likely reflects healthcare system differences (e.g., reimbursement criteria and treatment initiation thresholds) rather than underlying biological differences. However, given that this feature ranks highly in the predictive model, it raises questions regarding the transportability and generalizability of the model across healthcare systems with different treatment access patterns. To better understand whether this signal reflects underlying disease biology versus healthcare system structure, the authors may consider performing sensitivity analyses, such as retraining the model excluding geographic variables, or evaluating model performance separately within UK and non-UK subgroups. Such analyses would help clarify whether model performance is driven primarily by clinical predictors versus system-level factors and would strengthen confidence in the model’s generalizability.

Response: Thank you for these thoughtful insights. We agree that these are important considerations for model transportability; however, these analyses are not in scope with the current study. Therefore, we have included these considerations as directions for future research in the Discussion:

Lines 368-372: Future research could further explore the influence of healthcare system-level factors on model performance and generalizability, for example through sensitivity analyses excluding geographic variables or through subgroup evaluations across regions, to better distinguish the impact of clinical characteristics from structural differences in treatment access.

3. The conclusion section appears somewhat descriptive and could be more specific in terms of the clinical implications of the findings. Currently, mepolizumab is prescribed primarily for patients with severe asthma, and in clinical practice, pre-exposure CSE frequency is already an important component of treatment decision-making. In this context, it would be helpful if the authors could more clearly articulate how the proposed modeling approach provides additional value beyond existing clinical decision frameworks. In particular, the authors may consider clarifying how confident they are that these findings could support a more personalized medicine approach in real-world clinical practice, and to what extent the model could meaningfully influence treatment selection beyond current severity-based prescribing criteria. While strengthening the clinical interpretation would enhance the impact of the manuscript, I would like to clarify that I believe the scientific value of the study is not dependent on whether the model ultimately proves to be clinically actionable.

Response: Thank you for this comment. The following has been added to strengthen our Conclusion:

Lines 404-418: Notably, although several predictors identified in this analysis, such as pre-exposure CSE frequency, are already incorporated into current clinical decision-making frameworks, the application of ML methods enables the identification and integration of less well-established predictors and their interactions. The use of SHAP-based interpretability provided transparent identification and ranking of key drivers of response, with clear insights into the relative importance and, where applicable, the directionality of individual predictors. Importantly, the consistency of core features, including pre-exposure CSE frequency, baseline ACQ-5 score, BMI, activity impairment, and blood eosinophil counts across multiple endpoints supports the robustness of these findings. From a clinical perspective, the primary value of this approach lies in its ability to differentiate between patients who already meet treatment criteria. Even among individuals with similar exacerbation histories, the model integrates additional baseline characteristics to highlight meaningful differences in the likelihood of response. This more nuanced risk stratification may help inform treatment expectations and support more individualized management decisions in real-world practice. Overall, insights from this study pave the way for the development of predictive models that can inform and expedite more precise clinical decision-making, ultimately improving patient outcomes in the real-world management of severe asthma.

Reviewer #2: The manuscript is good and worse publishing

The article presents a robust application of machine learning (ML) methodologies to identify real-world predictors of mepolizumab response in patients with severe asthma using the REALITI-A dataset. The study is well-designed, utilizing advanced interpretability tools like SHAP to bridge the gap between complex model outputs and clinical utility.

Typographical Errors:

The manuscript is generally written in standard English and is intelligible. However, several minor errors and inconsistencies should be addressed to meet the journal's standard for clear and correct language. Here are some examples: On page 2, GSK plc is incorrectly written as GSK pic. On page 7, the data availability section contains a placeholder error: replace any insta instances of........

Response: Thank you for pointing these out. These errors have been corrected.

Data Availability:

The authors note that data access is restricted and available upon request. Given the journal's policy, the authors should ensure this level of restriction is fully justified by legal or ethical constraints.

Response: Our Data availability statement has been updated as follows: Please refer to GSK weblink to access GSK’s data sharing policies and as applicable seek anonymized subject level data via the link https://www.gsk-studyregister.com/, for this study (204710). This article reports secondary analyses using those existing anonymized data and does not generate any new patient-level datasets.

Conflict Disclosure:

The authors have clearly disclosed that several researchers are employees and shareholders of GSK, the manufacturer of mepolizumab. This transparency is essential given the potential for perceived bias.

Response: We have updated our Competing Interests Statement and Funding Statement to meet the journal’s requirements of transparency.

Competing Interests Statement: This study was funded by GSK (215038). Rafael Alfonso-Cristancho, Lingjiao Zhang, Peter Howarth and Koyo Usuba are employees and shareholders of GSK. Xinyang Liu, Tim Tian Yu Han, Natalie Nightingale and Ali Tehrani are employees of IQVIA. This commercial affiliation does not alter our adherence to PLOS ONE policies on sharing data and materials.

Funding Statement: This research was supported by GSK plc through grant number 215038. The funding agency provided support in the form of salaries for authors Rafael Alfonso-Cristancho, Lingjiao Zhang, Peter Howarth and Koyo Usuba, who are employees and shareholders of GSK plc. GSK plc paid consulting fees to IQVIA for study design, data analysis, and manuscript development services. Authors Xinyang Liu, Tim Tian Yu Han, Natalie Nightingale and Ali Tehrani are employees of IQVIA. The specific roles of the authors are articulated in the ‘author contributions’ section. The funder provided support in the form of salaries for authors (RA, LZ, PH, and KU), but had no additional role in study design, data collection and analysis, decision to publish, or preparation of the manuscript beyond the employment of the authors and the provision of the grant and contracting of IQVIA consulting services. No additional external funding was received for this study.

Attachment Submitted filename: Predictor of Nucala Response_PLOS One_Responses_v2.0.docx

# Peer review: Decision Letter 1

**DOI:** 10.1371/journal.pone.0362402.r003

**Authors:** Andre van Wijnen

25 Oct 2026

Machine learning approaches for the identification of real-world predictors of mepolizumab response

PONE-D-25-42854R1

Dear Dr. Usuba,

We’re pleased to inform you that your manuscript has been judged scientifically suitable for publication and will be formally accepted for publication once it meets all outstanding technical requirements.

Within one week, you’ll receive an e-mail detailing the required amendments. When these have been addressed, you’ll receive a formal acceptance letter and your manuscript will be scheduled for publication.

An invoice will be generated when your article is formally accepted. Please note, if your institution has a publishing partnership with PLOS and your article meets the relevant criteria, all or part of your publication costs will be covered. Please make sure your user information is up-to-date by logging into Editorial Manager at Editorial Manager® and clicking the ‘Update My Information' link at the top of the page. For questions related to billing, please contact billing support.

If your institution or institutions have a press office, please notify them about your upcoming paper to help maximize its impact. If they’ll be preparing press materials, please inform our press team as soon as possible -- no later than 48 hours after receiving the formal acceptance. Your manuscript will remain under strict press embargo until 2 pm Eastern Time on the date of publication. For more information, please contact onepress@plos.org.

Kind regards,

Andre van Wijnen

Academic Editor

PLOS One

Additional Editor Comments (optional):

Reviewers' comments:

Reviewer's Responses to Questions

**Comments to the Author**

Reviewer #2: All comments have been addressed

**********

2. Is the manuscript technically sound, and do the data support the conclusions?

Reviewer #2: Yes

**********

3. Has the statistical analysis been performed appropriately and rigorously?

Reviewer #2: Yes

**********

4. Have the authors made all data underlying the findings in their manuscript fully available?

The PLOS Data policy

Reviewer #2: Yes

**********

5. Is the manuscript presented in an intelligible fashion and written in standard English?

Reviewer #2: Yes

**********

Reviewer #2: The author responded well to the comments in my previous review.

I recommonend that this manuscript to be accepted and to proceed to the next step

**********

what does this mean?). If published, this will include your full peer review and any attached files.

If you choose “no”, your identity will remain anonymous but your review may still be made public.

**Do you want your identity to be public for this peer review?**  For information about this choice, including consent withdrawal, please see our Privacy Policy

Reviewer #2: **Yes:**  Walyeldin Elfakey

**********

# Editor report: Acceptance letter

**DOI:** 10.1371/journal.pone.0362402.r004

**Authors:** Andre van Wijnen

PONE-D-25-42854R1

PLOS One

Dear Dr. Usuba,

I'm pleased to inform you that your manuscript has been deemed suitable for publication in PLOS One. Congratulations! Your manuscript is now being handed over to our production team.

At this stage, our production department will prepare your paper for publication. This includes ensuring the following:

* All references, tables, and figures are properly cited

* All relevant supporting information is included in the manuscript submission,

* There are no issues that prevent the paper from being properly typeset

You will receive further instructions from the production team, including instructions on how to review your proof when it is ready. Please keep in mind that we are working through a large volume of accepted articles, so please give us a few days to review your paper and let you know the next and final steps.

Lastly, if your institution or institutions have a press office, please let them know about your upcoming paper now to help maximize its impact. If they'll be preparing press materials, please inform our press team within the next 48 hours. Your manuscript will remain under strict press embargo until 2 pm Eastern Time on the date of publication. For more information, please contact onepress@plos.org.

You will receive an invoice from PLOS for your publication fee after your manuscript has reached the completed accept phase. If you receive an email requesting payment before acceptance or for any other service, this may be a phishing scheme. Learn how to identify phishing emails and protect your accounts at https://explore.plos.org/phishing.

If we can help with anything else, please email us at customercare@plos.org.

Thank you for submitting your work to PLOS One and supporting open access.

Kind regards,

PLOS One Editorial Office Staff

on behalf of

Dr. Andre van Wijnen

Academic Editor

PLOS One
